# Do Quality Improvement Initiatives Improve Outcomes for Patients in Antiretroviral Programs in Low- and Middle-Income Countries? A Systematic Review

**DOI:** 10.1097/QAI.0000000000002085

**Published:** 2019-05-29

**Authors:** Sally Hargreaves, Keiran Rustage, Laura B. Nellums, Joshua E. Bardfield, Bruce Agins, Pierre Barker, M. Rashad Massoud, Nathan P. Ford, Meg Doherty, Gillian Dougherty, Satvinder Singh

**Affiliations:** a The Insitute for Infection and Immunity, St George's, University of London, London, United Kingdom;; b International Health Unit, Section of Infectious Diseases and Immunity, Imperial College London, London, United Kingdom;; c Healthqual, Institute for Global Health Sciences, University of California San Francisco, San Francisco, CA;; d URC, Chevy Chase, MD; e Institute for Healthcare Improvement, Boston, MA; f LLC/USAID ASSIST Project, University Research, Co, Bethesda, MD;; g Department of HIV/AIDS, World Health Organization, Geneva, Switzerland; and; h ICAP, Columbia University.

**Keywords:** HIV, AIDS, quality improvement, quality assurance, ART, LMICs

## Abstract

There have been a range of quality improvement (QI) and quality assurance initiatives in low- and middle-income countries to improve antiretroviral therapy (ART) treatment outcomes for people living with HIV. To date, these initiatives have not been systematically assessed and little is known about how effective, cost-effective, or sustainable these strategies are in improving clinical outcomes.

**Methods:**

We conducted a systematic review adhering to PRISMA guidelines (PROSPERO ID: CRD42017071848), searching PubMed, MEDLINE, Embase, Web of Science, and the Cochrane database of controlled trials for articles reporting on the effectiveness of QI and quality assurance initiatives in HIV programs in low- and middle-income countries in relation to ART uptake, retention in care, adherence, viral load suppression, mortality, and other outcomes including cost-effectiveness and long-term sustainability.

**Results:**

One thousand eight hundred sixty articles were found, of which 29 were included. QI approaches were categorized as follows: (1) health system approaches using QI methods; (2) QI learning networks including collaboratives; (3) standard-based methods that use QI tools to improve performance gaps; and (4) campaigns using QI methods. The greatest improvements were seen in ART uptake [median increase of 14.0%; interquartile range (IQR) −9.0 to 29.3], adherence [median increase of 22.0% (IQR −7.0 to 25.0)], and viral load suppression [median increase 26.0% (IQR −8.0 to 26.0)].

**Conclusions:**

QI interventions can be effective in improving clinical outcomes; however, there was significant variability, making it challenging to identify which aspects of interventions lead to clinical improvements. Standardizing reporting and assessment of QI initiatives is needed, supported by national quality policies and directorates, and robust research.

## Abstract 2

Supplemental Digital Content is Available in the Text.

## INTRODUCTION

Considerable strides have been made in the scale-up of antiretroviral therapy (ART) in low- and middle-income countries (LMICs). By the end of 2017, 21.7 million people living with HIV (PLHIV) were receiving ART, with a reported fall of 48% in AIDS-related deaths since a peak in 2005.^1,2^ However, only 75% of the estimated 36.7 million PLHIV globally know their status; 59% were receiving ART; and 47% were virologically suppressed. In LMICs with the highest burden of HIV, coordinated action is urgently needed to achieve global targets, so 90% of all PLHIV know their status, 90% of those diagnosed as HIV positive start ART, and 90% of all people receiving ART have durable viral suppression.^3^

**FIGURE 1.**
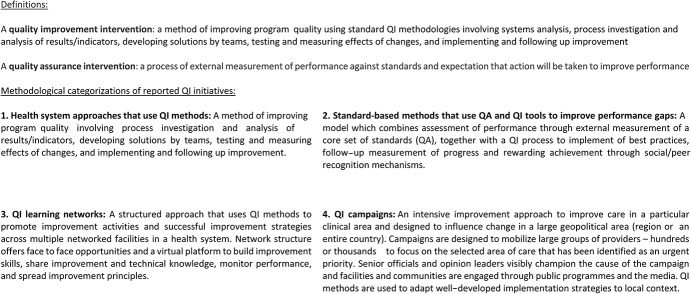
Definitions and categorization of QI/QA interventions used for this systematic review.

There is increasing recognition of gaps along the cascade of care and the need to strengthen the quality of service delivery.^4,5^ Various quality improvement (QI) and quality assurance (QA) strategies have been implemented in ART programs.^6,7^ Many definitions of QI exist in the literature. The recently published WHO National Quality and Policy Strategy Manual (http://apps.who.int/iris/bitstream/handle/10665/272357/9789241565561-eng.pdf?ua=1) defines QI as “a change in process in a health-care system, service, or supplier for the purposes of increasing the likelihood of optimal clinical quality of care measured by positive health outcomes for individuals and populations.”^8^ For this review, we consulted with the Quality of HIV Care Technical Working Group to generate a narrower definition (Fig. 1): “a method of improving program quality using standard QI methodologies involving system analysis, process investigation and analysis of results/indicators, developing solutions by teams, testing and measuring effects of changes, and implementing and following up improvement.” QA is defined as “a process of external measurement of performance against standards and expectation that action will be taken to improve performance” (Fig. 1).

Across the global literature on QI initiatives globally, there is a high risk of bias, with studies predominantly from high-income countries or not specifically focused on HIV treatment programs.^9^ Furthermore, the evidence base is limited by a lack of systematic or robust examination of evidence on the effectiveness of initiatives to improve quality in the delivery of ART programs. As a result, there is a lack of consensus on effective, cost-effective, or sustainable approaches.

We undertook a systematic review to identify and synthesize evidence base on the cost-effectiveness, acceptability, impact of QI and QA initiatives on key clinical outcomes in ART programs in LMICs, and wider indicators.

## METHODS

This systematic review adhered to the Preferred Reporting Items for Systematic Reviews and Meta-Analyses (PRISMA) guidelines,^10^ and the protocol published on the PROSPERO database (CRD42017071848; https://www.crd.york.ac.uk/prospero/display_record.php?RecordID=71848).

### Inclusion and Exclusion Criteria

We included randomized controlled trials, observational studies, and gray literature reporting on the effectiveness of QI and QA initiatives implemented in ART programs in LMICs as defined by the World Bank classification.^11^

We considered evidence of effectiveness for predefined outcomes including:

- Key outcomes: ART uptake, retention in care, adherence, viral load suppression, and mortality
- Other outcomes: uptake of screening, identification, and treatment of incident opportunistic infections, process indicators such as CD4/VL testing, acceptability to patients and/or service providers, and prevention of mother-to-child HIV transmission
- Cost-effectiveness
- Wider impact (eg, long-term sustainability)

There were no restrictions on language. Research was excluded if it was conducted in high-income countries, data for key outcomes were not present, or the main focus of the intervention was task-shifting, which is not considered a QI or QA intervention for the purposes of this review. In addition, articles only reporting on specific types of clinical care/interventions (eg, prevention of mother-to-child HIV transmission) were not included. These studies represent a substantial and important literature, and merit separate reviews tailored to the specific methodologies and outcomes relevant to these distinct areas. Training, clinical mentoring, and supportive supervision, without the precise improvement actions defined as part of QI, were not included because the focus was on QI methods rather than the specific components of interventions; in addition, we note that supportive supervision and mentorship has its own literature, as part of broader QA efforts. These decisions were made in consultation with the Quality of HIV Care Technical Working Group.

### Search Strategy

We searched the databases PubMed, MEDLINE, Embase, Web of Science, and the Cochrane database of controlled trials from inception to October 24, 2017. We used a Boolean search strategy with keywords relevant to QI, QA, and HIV, identified from relevant research, previous related systematic reviews, and consultation with the World Health Organization and Quality of Clinical HIV Care Technical Working Group (see Supplemental Digital Content, http://links.lww.com/QAI/B330). Gray literature was obtained through a hand-search of web search engines, related websites, and submission by key experts in the Quality of Clinical HIV Care Technical Working Group. These experts were also formally invited to submit the gray literature (conference abstracts, unpublished reports, presentations, etc) relating to QI in ART programs in LMICs. The bibliographies of included articles were cross-referenced, and key experts consulted, to identify additional research.

### Data Extraction and Analysis

Title and abstract screening was conducted by 3 reviewers (K.R., L.B.N., and S.H.). Subsequent full-text screening was conducted by 2 reviewers (S.H. and K.R.). Discrepancies were resolved through discussion with a third reviewer. Screening was facilitated by the web-based application Rayyan.^12^

Data were extracted by 2 reviewers (K.R. and S.H.) using a piloted form on predefined outcomes determined by the study team through consultation with experts in the field. The outcomes included the following: ART uptake, retention in care, adherence, viral load suppression, mortality, and other wider outcomes (opportunistic infections, acceptability to patients and service providers, process indicators, cost-effectiveness, and long-term sustainability).

Quality and risk of bias were assessed by 2 reviewers (K.B. and L.N.) using a piloted critical appraisal tool that included indicators from the Joanna Briggs and Newcastle–Ottawa scales as relevant for the diverse study types.^13,14^ The average quality score was 69.1%. Quality scores were divided into tertiles, categorizing articles in relation to whether they were scored in the low (22.7%–59.1%), medium (63.6%–72.7%), or high (77.3%–100%) third of articles. Although study quality was assessed to indicate methodological rigor and clarity and transparency in reporting, studies were not excluded on the basis of quality to strengthen the transparency of the review and comprehensively report on all available evidence, including both primary peer-reviewed research and the gray literature.

All included studies were categorized in relation to methodological approaches agreed in collaboration with the identified panel of experts (Fig. 1): health system approaches using QI methods, standard-based methods that use QI tools to improve performance gaps, campaigns that use QI methods, and QI learning networks including collaboratives.

Summary analyses were conducted in Microsoft Excel and Stata 15 to show the distribution of percent increase reported for each outcome by study and to calculate the mean and median increase in the percentage of patients with each clinical outcome to provide an indication of the reported impact of QI interventions across the available evidence base. We also compared the median increase in ART uptake by methodological approach and country setting and in programs focused on the prevention of mother-to-child transmission (PMTCT) compared with the general patient population. Where relevant data were not reported for the predefined outcomes, articles were not included in the syntheses.

### Role of the Funding Source

The funders of the study had no role in the study design, data collection, data analysis, data interpretation, writing of the report, or the decision to submit the article for publication. All authors had full access to all data and responsibility for the decision to submit for publication.

## RESULTS

### Overview of the Included Literature

**FIGURE 2.**
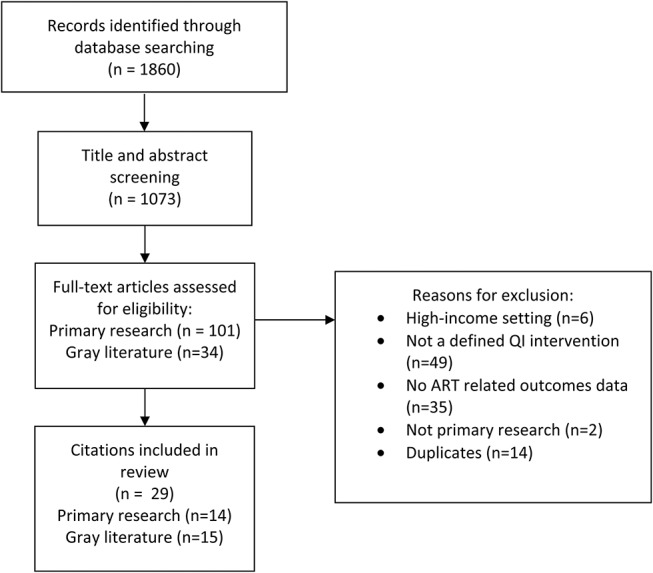
PRISMA flow diagram.

A total of 1860 records were identified in the database searches, with 1073 publications being subject to title and abstract screening after removing duplicates. One hundred one publications were carried forward for full-text screening, in addition to 34 gray literature records. Of the 135 articles included in the full-text screening, 29 were included in the review (Fig. 2), including 14 peer-reviewed^15–28^ and 15 gray literature articles (see Table 1, Supplemental Digital Content, http://links.lww.com/QAI/B330).^29–43^ There was a significant variation in the quality of included studies (see Table 1, Supplemental Digital Content, http://links.lww.com/QAI/B330), with limited detail in relation to measures, outcomes or definitions used, observational designs across most studies, and a lack of data to isolate the effects of interventions.

Many initiatives involved a multifaceted package of interventions. No studies were identified that reported solely on QA; therefore, our results present no data on QA initiatives. There are a range of quality initiatives being evaluated in LMICs [Standards-based Management and Recognition, HIVQUAL/HEALTHQUAL (Note HIVQUAL changed its name to HEALTHQUAL), Breakthrough Series Collaborative, and ASSIST], as well as multiple other approaches (quality collaboratives, performance management–QI, mentorship, and training without a specific QI component, among others).

**FIGURE 3.**
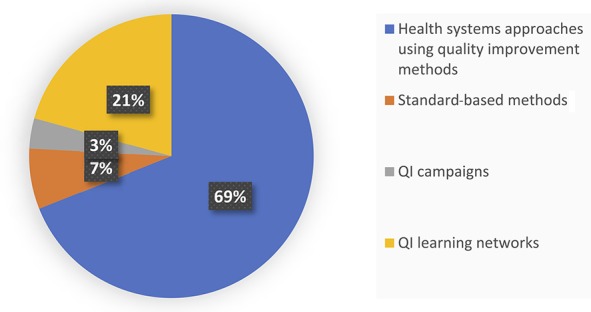
Proportion of QI initiatives reported in the included literature.

**FIGURE 4.**
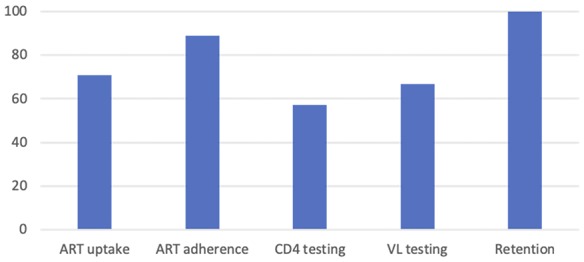
Proportion of included studies reporting improvements in key outcomes.

We grouped approaches into 4 main categories with support from experts in the Quality of HIV Care Technical Working Group (Fig. 1): health systems approaches using QI methods (n = 20),^21,22,24–26,29–43^ standard-based methods that use QI tools to improve performance gaps (n = 2),^17,23^ campaigns that use QI methods (n = 1),^28^ and QI learning networks including collaboratives (n = 6).^15,16,18–20,27^ Initiatives were performed in 13 LMICs, including South Africa,^15,19,20,28^ Vietnam,^16,35^ Zambia,^17,23^ Nigeria,^18,27^ Uganda,^21,22,31,36,40^ Mozambique,^21,22^ Namibia,^21,22,29,34,42,43^ Haiti,^21,22,24,32,36,41^ Thailand,^25,26^ Nicaragua,^33^ Kenya,^38^ Tanzania,^37^ and Guyana,^34,39^ (Fig. 3). Across the studies, improvements were reported for ART uptake (n = 17), ART adherence (n = 10), CD4 testing (n = 6), retention (n = 5), and cost (n = 1) (Fig. 4).

### Overall Impact of QI Approaches

**FIGURE 5.**
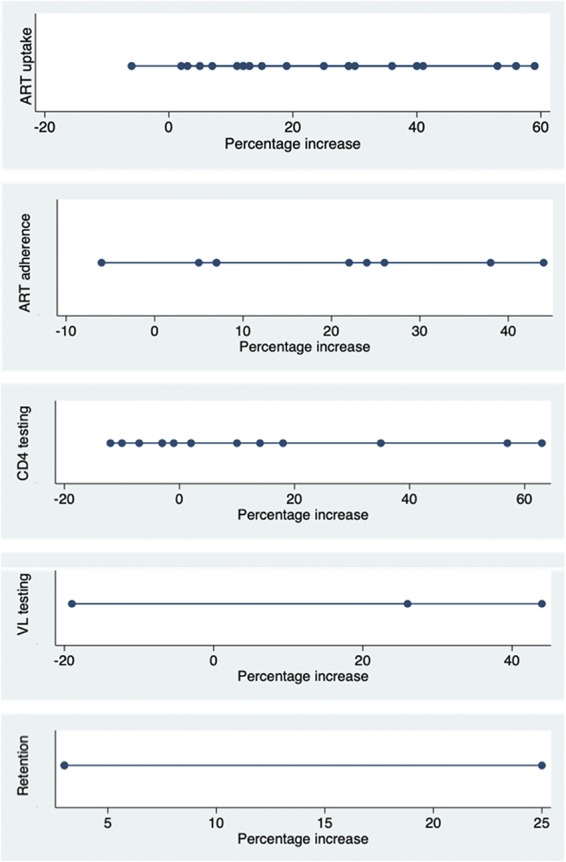
Median percentage increase in outcomes across QI/QA initiatives.

There was significant variation across the evidence regarding the influence of QI initiatives on clinical outcomes (Fig. 5). The greatest improvement was seen in ART uptake median increase of 14.0%; interquartile range (IQR) −9.0 to 29.3 of patients across sites], ART adherence [median increase of 22.0% (IQR −7.0 to 25.0)], and viral load suppression [median increase 26.0% (IQR −8.0 to 26.0)] (see Table 1, Supplemental Digital Content, http://links.lww.com/QAI/B330).

These outcomes should be considered in the context of other initiatives and national policy and program changes that may have targeted ART uptake and adherence, and viral load suppression at the same time. Improvements may also be partly attributed to inclusion of programs focused on the prevention of mother-to-child transmission (PMTCT; see Table 1, Supplemental Digital Content, http://links.lww.com/QAI/B330). We conducted a sensitivity analysis to examine the increase in ART uptake in pregnant women compared with the general patient population across the evidence, identifying that the median percentage increase in patients initiating ART in programs focused on PMTCT was 19.0% (IQR 13.5–40.5) compared with 13.0% (IQR 4.5–29.3) for programs directed at the general population (Table 1), although this was not statistically significant.

**TABLE 1.**
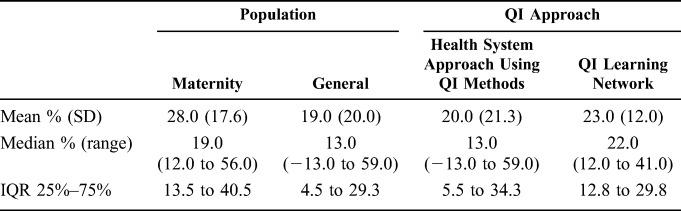
Changes in ART Uptake by Population and QI/QA Approach

There was also significant variation in the effectiveness of QI interventions across initiatives implemented in different country settings. The highest median percentage improvement in ART uptake was seen in Vietnam (29.0%, IQR 29.0–29.5), followed by Haiti (25.0%, IQR 13.0–37.0); however, there were limited data for other countries and variability in the length of follow-up, making it challenging to make meaningful cross-country comparisons (Table 2). For example, 3 studies looking to improve ART uptake had follow-up periods of 8,^35^ 12,^31^ and 24 months.^32^

**TABLE 2.**
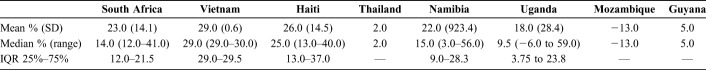
Changes in ART Uptake by Country of Study

### Effectiveness by QI Methodological Approach

#### Health System Approaches Using QI Methods

Twenty studies (5 published and 15 gray literature) were categorized as health system approaches using QI methods (eg, including systems analysis, process investigation and analysis of results/indicators, developing solutions by teams, testing and measuring effects of changes, and implementing and following up improvement) (Fig. 1). Fourteen studies reported outcomes related to ART uptake, generally showing a positive impact. In 1 study, the number of eligible children taking up treatment rose from 12% to 25%,^32^ whereas ART uptake rose from 61% to 90% in another health system intervention.^35^ Even in a previously well-performing setting, ART uptake rose from 98% to 100% after the introduction of the intervention.^25^ However, there were also 2 examples of a decline in ART uptake. In one such case, uptake fell from 82% to 76% over a 12-month period,^31^ whereas in another, CD4 testing for subsequent ART treatment in individuals with CD4 cell counts <200/µL dropped from 100% to 90% of eligible individuals.^26^ Overall, the median percentage increase in patients initiated on ART in programs using this methodological approach was 13.0% (IQR 5.5–34.3), with a range of −13.0% (decrease) to 59.0% (Table 1).

Among articles reporting health system approaches using QI methods, there were also 9 studies reporting ART adherence (2 published and 7 gray literature) and 5 studies reporting on retention. Six of the 9 studies reporting adherence outcomes showed improved adherence documentation and recording procedures, as opposed to being related directly to the level of adherence seen in individuals on treatment. One study reported that pediatric adherence rose from 43% to 81%,^36^ whereas a multicenter study documented improvements in adherence from 90% to 97% in Namibia, 63%–85% in Uganda, 66%–97% in Mozambique, 29%–83% in Haiti, and 56%–80% in Guyana.^39^ One study reported a decrease in adherence from 82% to 76%; however, this finding may be due to an associated improvement in documentation procedures and adherence assessment, which increased from 81% to 92%.^31^ Retention was also suggested to slightly increase across the studies (median increase 3.0%, IQR 3.0–3.0; range 3.0–25.0).

Health system approaches using QI methods were also associated with positive impacts on process indicators. CD4 testing rates over 6 months improved from 43% to 78% in 1 setting^35^ and from 10.8% to 20.5% when testing individuals on enrollment and after 6 months of follow-up.^41^ However, there were not always substantial gains or improvements.^25^ Prophylaxis access for opportunistic infections also increased. One study reported an increase from 12% to 95% of children receiving cotrimoxazole prophylaxis,^43^ whereas another reported that prophylaxis peaked during the intervention at 84.8% of eligible individuals receiving treatment.^41^ Tuberculosis screening also increased across all reports, with 1 example citing an increase from 24% to 99%.^26^

#### QI Learning Networks Across Multiple Sites (Including Collaboratives)

Six peer-reviewed articles reported on QI learning networks (including collaboratives), which included coaching and mentoring of health care staff, alongside peer exchange to address performance gaps. One model adopted was the Breakthrough Series (BTS) collaborative,^15^ which brings facility teams together to learn QI methods, identify performance gaps, and plan implementation interventions, with follow-up visits from quality mentors to coach teams on using QI methods and maintain momentum for improvement. Four studies reported on ART uptake outcomes, all of which showed improvements. Overall, the median increase in ART uptake was 22.0% (IQR 12.8–29.8) (Table 1). One study reported a district level increased uptake of 62%–91% over 30 days.^16^ In another setting, monthly ART initiation rose 185.5% after initiation of the intervention, from 179 initiations per month to 511.^19^ ART adherence and retention were only reported in 1 study each. The study that included adherence outcomes reported improvements in documentation of adherence support and adherence assessment procedures, and it was not clear if there was an impact on patient adherence. In this study, documentation improved from 83% to 99% at the provincial level and from 54% to 97% at the district level.^16^ In terms of ART retention, the 1 reporting study found no impact associated with the intervention, with no statistically significant difference in retention of postpartum women after 6 months in the intervention and control arms.^18^

The effects of QI interventions on process indicators, opportunistic infection, and TB screening were sparsely reported. The studies reporting on process indicators indicated an improvement associated with the intervention. CD4 testing in the previous 6 months increased from 80% to 94% at the provincial level and from 72% to 74% at the district level in 1 setting,^16^ and rates of early infant testing at 4–6 weeks increased in intervention sites from 25.3% to 48.8% in another study.^18^ Data quality was reported to have improved in another setting.^27^ Cotrimoxazole prophylaxis prescription to combat opportunistic infections in eligible individuals increased in one study from 31% to 99% at the provincial level and from 75% to 83% at the district level, whereas TB screening in this same study also increased from 15% to 100% at the provincial level and from 18% to 79% at the district level.^16^

One study further looked at the acceptability of the intervention to health care workers and patients. However, despite enthusiastic participation in the program by health care workers, there was no measurable increase in patient satisfaction.^27^

#### Standard-Based Methods that Use QI Tools to Improve Performance Gaps

The 2 citations that used standard-based methods only reported on acceptability of the intervention (2 published and 0 gray literature).^17,23^ The studies initially sought to define performance criteria relevant to the context, before using outside support to measure pre-existing performance gaps (in relation to the new standards), which are subsequently addressed and reassessed. In both studies, the authors indicated that health care worker perceptions of their work environment were positively impacted by the intervention, whereas they declined in comparison sites. Standard-based methods also increased ART readiness scores and provider performance related to ART and PMTCT at the intervention sites versus comparison sites.^17^

#### Campaigns That Use QI Methods

Only 1 study reported a QI campaign among health workers (Fig. 1) in 2 districts in KwaZulu-Natal, South Africa, between 2009 and 2010, and was included as a separate category on consultation with the expert group.^28^ This study reported on outcomes related to ART uptake among HIV-infected pregnant women and cost, finding that monthly referrals for ART rose from 78.7 [95% confidence interval (CI): 69 to 89] individuals to 188.2 (95% CI: 167 to 209), whilst monthly ART initiation concurrently rose from 20.7 (95% CI: 2 to 40) individuals to 123.8 (95% CI: 108 to 140), with much smaller increases in the control district (39–55 initiations per month). In terms of cost, there was no formal analysis; however, the authors state that they believed the interventions could be rapidly implemented with a low incremental cost because no new personnel were added to the existing health system.

## DISCUSSION

This is the first systematic review to explore the evidence base on the effectiveness of interventions to improve quality in ART programs in LMICs and to define and categorize methodological approaches being implemented in these settings. The review suggests that QI initiatives at a site level, applied at multisite learning collaboratives, and as part of a campaign, were associated with increased ART uptake and ART adherence across sites. However, there was variability in the effect across studies and approaches. Although the findings point to the potential effectiveness of QI interventions to improve quality, we should interpret the results with caution given the limitations of the study designs; ie, lack of comparators or the contribution of other initiatives. This points to the need for more rigorous evaluation methods to assess the impact of QI interventions and innovative approaches to assessing quality in public health initiatives more broadly. It is also important to be aware of a bias toward publication of positive results.

The effect of other program factors is noted in some cases, both in rapid improvement of clinical outcomes and in an apparent worsening of outcomes in some cases, which could influence the interpretation of QI studies. For example, in Uganda, ART uptake decreased from 82% to 76% over 12 months,^31^ and in Thailand, CD4 testing coverage dropped from 100% to 90%.^26^ Because both of these studies were supported by authors of this article, further information was obtained from the program reports, and it is reported that in subsequent years, ART coverage increased, whereas the number of patients enrolled in care increased dramatically over that time period. As well, in Thailand, the drop in CD4 monitoring may have been related to a rapid expansion in the program from 12 hospitals in 2002 to 64 in 2006, thus highlighting the impact of other program factors. The heterogeneity across QI initiatives makes it challenging to discern the relative benefit of specific QI approaches. Both increases and decreases in outcomes need to be contextualized in relation to factors such as methodological variations, socioeconomic conditions, changes in health expenditure, and other possible factors such as stock-outs and documentation challenges.

Our findings concur with a recent systematic review exploring the QI collaborative approach mostly being evaluated in high-income countries, which reported that the evidence for effectiveness of initiatives that use QI collaboratives is positive, but that the effects cannot be predicted with great certainty with limited evidence of effectiveness, cost-effectiveness, and sustainability.^7^

Many QI initiatives involve a complex multifaceted package of interventions. For example, HEALTHQUAL is a public health model for capacity building and can involve multiple interventions as part of the effort to improve health systems. Studies may also report outcomes on initiatives involving QI and other capacity-building initiatives and also adapt established QI models to local use.^16^ This means that it is often not straightforward to identify which particular aspect of a QI initiative has led to improvements. While QI initiatives target multiple health systems components of clinical processes, it is challenging to identify which factors contribute to improvements, because other national initiatives may be introduced simultaneously making it challenging to identify which factors contribute to improvements. “However it is hypothesized that this more holistic approach required...” if possible to rephrase think would be better to say “It has been suggested that QI initiatives and methods should be judged on the overall effectiveness of the program,” rather than attempting to elucidate which specific components are most integral to the benefits observed.^44^

### Strengths and Limitations

The review used a rigorous approach, aligning with PRISMA guidelines and was registered prospectively in PROSPERO. The results are driven by a comprehensive search strategy, which includes 5 databases and gray literature. The assessment of key clinical outcomes alongside other wider effects allowed for a broad examination of evidence of effectiveness, enabling a robust synthesis to evaluate and inform QI initiatives. A formal meta-analysis was not deemed appropriate given the heterogeneity in QI approaches, study methods, and reporting. However, studies consistently reported percent increases in the predefined outcomes, allowing such data to be combined and summarized to provide an indication of the reported impact of QI interventions across the available evidence base.

The significant variation in the quality of study designs as well as reported outcomes and their definitions highlights the need to improve methodological rigor in evaluation and research in this area. The evidence base would be strengthened by efforts to adopt emerging QI evaluation designs.^45^ The decision to retain articles and not exclude on the basis of quality was essential to demonstrate the range of quality across the available evidence base and the need to strengthen study quality, benefitting the transparency and impact of this review.

There was limited reporting of QI initiatives in both the published and gray literature, with few peer-reviewed studies of the effectiveness of QI/QA initiatives, or publicly available evaluations. For many organizations, QI is part of standard technical assistance for countries, and reports of QI work may be disseminated only internally within the support organizations or funding bodies.

Another key limitation is that improvements may not be solely attributed to the QI interventions but also to wider initiatives targeting HIV programs or health care systems, or improvements in documentation and data recording procedures. For example, changes in national policies to shift CD4 eligibility criteria or HIV testing campaign or expansion of sites/task-shifting/decentralization effort would improve certain outcomes unrelated to the specific QI efforts. Future research should aim to address these concerns, and where interventions are implemented, these wider contextual factors should be acknowledged through the adoption of implementation science frameworks.

The WHO recently published recommendations for standard program reporting standards,^46^ standards for sexual and reproductive health, which could be enhanced with practitioners agreeing on a common set of clinical and health system outcomes. It has also been proposed that the design and evaluation of such programs should be mutually informing for both improvers and evaluators. The Framework for Learning about Improvement and the Evaluation Continuum, for example, provides a structure to improve the design, implementation, and evaluation of improvement programs. This could enable both the generation of more robust and generalizabile data and improve understandings of the mechanisms contributing to improvement and how to scale-up improvement efforts.^47^

Evaluation of programmatic quality could be further enhanced through improved reporting mechanisms to assess implementation, which could be based on commonly agreed core components for QI programs.^48^ Standardized quality scores based on national guidelines for care recommendations have been developed in similar contexts assessing adherence to such guidelines and have been shown to be associated with decreased mortality in these settings.^49^ The implementation of such procedures across all settings would enable a fuller comparison of QI programs, alongside their respective standards of quality both before and after intervention.^48^

The ability to assess the impact of QI interventions on clinical outcomes or determine which specific approaches result in the greatest improvements is also limited by observational study designs. However, a key strength of the approaches across the included studies is the development and implementation of interventions in the incorporating local context at which they are aimed, which is likely to increase the success of QI initiatives.^44^

Most of the included studies had externally funded implementing partners providing staff to support data collection, implementation, and evaluation. This reliance on external technical support raises a question about the sustainability and scalability of these interventions. Many interventions included participation from management and frontline teams of the local health system but did not demonstrate the ability to undertake QI efforts using local data systems, or sustainability or scalability beyond the life of the project.

The use of external or parallel data systems also circumvented the poor quality of routinely collected data—a major obstacle to the implementation, scale-up, sustainability, and evaluation of QI interventions. Many of the studies reported in the review used data that were collected independently of the local routine data reporting systems, and the future performance of those initiatives may be dependent on continued support. There are some examples of large scale QI programming that used routine data systems, showing that is possible to improve local data systems to the point where they can be used for QI approaches. Although one of the aims of this review was to assess sustainability, the identified literature did not specifically address this question, so no data are presented.

### Conclusions and Further Research

Our findings support the effectiveness of QI in ART programs, with the greatest improvement in clinical outcomes seen in ART uptake, adherence, and viral load suppression, and in programs focused on PMTCT. Although the evidence suggests QI initiatives are associated with improved clinical outcomes, there was significant heterogeneity across approaches, settings, and reporting, making it challenging to identify best practices and to understand what specific aspects of these interventions lead to significant and sustainable clinical improvements in an LMIC context. Furthermore, there was very limited evidence on the cost-effectiveness of these interventions and a need for formal economic analyses to determine the cost implications of QI initiatives.

The findings point to the need to better use standard evaluation designs and reporting methods.^46,50^ Although programmatic quality reporting focusing on the implementation of interventions against established guidelines would enhance our ability to compare the effectiveness of interventions across diverse settings, it could also be tailored the specific context in which programs are being delivered, for example, primary health care sites as opposed to specialty care.^5,48,49,51^

A further key consideration for practice is the need to embed QI initiatives within national efforts to improve health systems. Most of the studies in this review were supported or conducted by implementing partners with external funding, using external data collection and analysis systems. The question remains what approaches will be needed to support the capacity of health systems in LMICs to undertake these approaches through their existing quality management infrastructure without a need for external partners and funding. In a related field, the WHO-led Network for Improving Quality of Care for Maternal Newborn and Child Health Care is driving the approach of moving care away from NGO-led, fragmented, and often unsustainable individual QI projects toward country-led initiatives.^52^ WHO is also providing guidance to governments on how to design and organize national QI programming through development of their national quality policy and strategies, an approach that supports embedding QI in ART programmes within the context of a national quality policy and strategy in support of broader universal health coverage goals^53,54^ across HIV programs globally.^52^

Research is needed to evaluate the effectiveness, cost-effectiveness, and long-term sustainability of QI interventions and to identify which elements contribute to improved clinical outcomes in LMICs. This will support efforts to achieve the 90-90-90 goals^55^ and aligns with the recent Lancet Global Health Commission on High-Quality Health in the SDG Era.^56^ Ultimately, evidence-based QI methods must be integrated into broader efforts to ensure the delivery of high-quality care in low resource settings, within the context of the WHO Framework on integrated people-centered health services.^57,58^

## References

[1] UNAIDS. Global AIDS Update 2016. 2016 Available at: http://www.unaids.org/sites/default/files/media_asset/global-AIDS-update-2016_en.pdf. Accessed February 21, 2018.

[2] UNAIDS. Fact Sheet—Latest Global and Regional Statistics on the Status of the AIDS Epidemic. 2018 Available at: http://www.unaids.org/sites/default/files/media_asset/UNAIDS_FactSheet_en.pdf. Accessed February 21, 2018.

[3] UNAIDS. 90-90-90: An Ambitious Treatment Target to Help End the AIDS Epidemic. Geneva, Switzerland 2014 Available at: http://www.unaids.org/sites/default/files/media_asset/90-90-90_en.pdf. Accessed February 21, 2018.

[4] UNAIDS. Ending AIDS: Progress towards the 90-90-90 Targets. Geneva, Switzerland 2017 Available at: http://www.unaids.org/sites/default/files/media_asset/Global_AIDS_update_2017_en.pdf. Accessed March 26, 2018.

[5] The Lancet Global Health. Are we ready for a quality revolution? Lancet Glob Heal. 2018;6:e121. 10.1016/S2214-109X(17)30500-4 29389526

[6] Joint United Nations Programme on HIV/AIDS (UNAIDS). On the Fast-Track to End AIDS: UNAIDS 2016–2021 Strategy. Geneva, Switzerland 2016 Available at: http://www.unaids.org/sites/default/files/media_asset/20151027_UNAIDS_PCB37_15_18_EN_rev1.pdf. Accessed January 17, 2018).

[7] Schouten LMT Hulscher MEJL van Everdingen JJE Evidence for the impact of quality improvement collaboratives: systematic review. BMJ. 2008;336:1491–1494. 18577559 10.1136/bmj.39570.749884.BE PMC2440907

[8] World Health Organization. A Handbook for National Quality Policy and Strategy: A Practical Approach for Developing Policy and Strategy to Improve Quality of Care. Geneva, Switzerland: WHO; 2018.

[9] Buckley GJ Pittluck RE Improving Quality of Care in Low- and Middle-Income Countries: Workshop Summary. Geneva, Switzerland: WHO; 2015. 10.17226/21736. 26677493

[10] Moher D Liberati A Tetzlaff J; PRISMA Group TP. Preferred reporting items for systematic reviews and meta-analyses: the PRISMA statement. PLoS Med. 2009;6:e1000097. 19621072 10.1371/journal.pmed.1000097 PMC2707599

[11] The World Bank. World Bank Country and Lending Groups—World Bank Data Help Desk. Available at: https://datahelpdesk.worldbank.org/knowledgebase/articles/906519-world-bank-country-and-lending-groups. Accessed February 21, 2018.

[12] Ouzzani M Hammady H Fedorowicz Z Rayyan—a web and mobile app for systematic reviews. Syst Rev. 2016;5:210. 27919275 10.1186/s13643-016-0384-4 PMC5139140

[13] Wells G Shea B O'Connel D The Newcastle-Ottawa Scale (NOS) for Assessing the Quailty of Nonrandomised Studies in Meta-Analyses. Ottowa, Canada: University of Ottowa; 2009.

[14] Joanna Briggs Institute. The Joanna Briggs Institute Critical Appraisal Tools for Use in JBI Systematic Reviews—Checklist for Case Series. 2016 Available at: http://joannabriggs.org/research/critical-appraisal-tools.html. Accessed August 8, 2017.

[15] Barker P Barron P Bhardwaj S The role of quality improvement in achieving effective large-scale prevention of mother-to-child transmission of HIV in South Africa. AIDS. 2015;29:S137–S143. 26102624 10.1097/QAD.0000000000000718

[16] Cosimi LA Dam HV Nguyen TQ Integrated clinical and quality improvement coaching in Son La Province, Vietnam: a model of building public sector capacity for sustainable HIV care delivery. BMC Health Serv Res. 2015;15:269. 26184505 10.1186/s12913-015-0935-8 PMC4504451

[17] Kols A Kim YM Bazant E A standards-based approach to quality improvement for HIV services at Zambia Defence Force facilities: results and lessons learned. AIDS. 2015;29:S145–S153. 26102625 10.1097/QAD.0000000000000720

[18] Oyeleledun B Oronsaye F Oyelade T Increasing retention in care of HIV-positive women in PMTCT services through continuous quality improvement-breakthrough (CQI-BTS) series in primary and secondary health care facilities in Nigeria: a cluster randomized controlled trial. The Lafiyan Jikin. J Acquir Immune Defic Syndr. 2014;67:S125–S131. 25310118 10.1097/QAI.0000000000000320

[19] Webster P Sibanyoni M Malekutu D Using quality improvement to accelerate highly active antiretroviral treatment coverage in South Africa. BMJ Qual Saf. 2012;21:315–324. 10.1136/bmjqs-2011-000381 PMC3311871 22438327

[20] Youngleson MS Nkurunziza P Jennings K Improving a mother to child HIV transmission programme through health system redesign: quality improvement, protocol adjustment and resource addition. PLoS One. 2010;5:e13891. 21085479 10.1371/journal.pone.0013891 PMC2976693

[21] Bardfield K Agins B Palumbo M Improving rates of cotrimoxazole prophylaxis in resource-limited settings: implementation of a quality improvement approach. Int J Qual Heal Care. 2014;26:613–622. 10.1093/intqhc/mzu085 25335758

[22] Massoud MR Shakir F Livesley N Improving care for patients on antiretroviral therapy through a gap analysis framework. AIDS. 2015;29:S187–S194. 26102630 10.1097/QAD.0000000000000742

[23] Bazant E Sarkar S Banda J Effects of a performance and quality improvement intervention on the work environment in HIV-related care: a quasi-experimental evaluation in Zambia. Hum Resour Health. 2014;12:1–11. 25528044 10.1186/1478-4491-12-73 PMC4290808

[24] Joseph JP Jerome G Lambert W Going beyond the vertical: leveraging a national HIV quality improvement programme to address other health priorities in Haiti. AIDS. 2015;29:S165–S173. 26102627 10.1097/QAD.0000000000000715

[25] Lolekha R Chunwimaleung S Hansudewechakul R Pediatric HIVQUAL-T: measuring and improving the quality of pediatric HIV care in Thailand, 2005–2007. Jt Comm J Qual Patient Saf. 2010;36:541–551. 21222356 10.1016/s1553-7250(10)36082-x

[26] Thanprasertsuk S Supawitkul S Lolekha R HIVQUAL-T: monitoring and improving HIV clinical care in Thailand, 2002-08. Int J Qual Heal Care. 2012;24:338–347. 10.1093/intqhc/mzs008 22665387

[27] Osibo B Oronsaye F Alo O Using small tests of change to improve PMTCT services in Northern Nigeria: experiences from implementation of a continuous quality improvement and breakthrough series program. J Acquir Immune Defic Syndr. 2017;75:S165–S172. 28498186 10.1097/QAI.0000000000001369

[28] Ngidi W Reddy J Luvuno Z Using a campaign approach among health workers to increase access to antiretroviral therapy for pregnant HIV-infected women in South Africa. J Acquir Immune Defic Syndr. 2013;63:133–139. 10.1097/QAI.0b013e318291827f 23514955

[29] Bardfield J Agins B Akiyama M A quality improvement approach to capacity building in low- and middle-income countries. AIDS. 2015;29:S179–S186. 26102629 10.1097/QAD.0000000000000719

[30] Bardfield J Agins B Palumbo M A Quality Improvement Approach to Scale-Up of ART in Resource Limited Settings. San Francisco, CA: University of California, San Francisco 1339.

[31] Behumbiize P Ssendiwala J Kayita G Organizational Structure and Capacity for Evaluating the Quality of Care Among HIV Clinics in Northern Uganda. New York, NY: New York State Department of Health; 2006.

[32] Bijou S Labbe C Nancy R Use of an Electronic Medical Record to Implement, Monitor, and Improve HEALTHQUAL Clinical Care Indicator Performance Rates. New York, NY: New York State Department of Health; 2005.

[33] Broughton E Nunez D Moreno I Cost-effectiveness of improving health care to people with HIV in Nicaragua. Nurs Res Pract. 2014;2014:1–6. 10.1155/2014/232046 PMC4058229 24977038

[34] HealthQual International. Performance Measurement Data Report 2015. San Francisco, CA: HealthQual International; 2015.

[35] HealthQual International. HIVQUAL Vietnam: National and Local Leadership Support for Improvement in HIV Care & Treatment. San Francisco, CA: HealthQual International; 2012.

[36] Kayita G Ssendiwala J Lamothe N Building a Sustainable National Model to Improve Pediatric and Maternal Health Care in Haiti and Uganda. Kampala, Uganda: Ministry of Health; 2011.

[37] Kimaro J Kihwele D Stover K Empowering Community Groups to Support Access and Retention in HIV Care in Muheza, Tanzania—Technical Report. Bethesda, MD: USAID ASSIST Project; 2015.

[38] Mohamed I Wanyungu J Abass M Improving Quality of Care for People Living with HIV and AIDS in Kenya: A Case of Coast Province. Nairobi, Kenya: CDC-Kenya; 2010:54.

[39] Palumbo M Birchard R Geis M A Public Health Approach to Building Sustainable National Quality Management Programs in Low- and Middle-Income Countries. New York, NY: New York State Department of Health AIDS Institute:8599.

[40] Ssendiwala J Kayita G Palumbo M National Scale up of Quality Improvement Activities : A Case Study of Uganda. Kampala, Uganda: Ministry of Health.

[41] Thimothe G Duval N Lauture D Application of a National Electronic Health Records System for Measuring Performance for Improvement in Systems of Care for Persons Living with HIV Measurement of Effect Changes. Port-au-Prince, Haiti: Ministry of Public Health and Population.

[42] USAID Applying Science to Strengthen and Improve Systems Project. Bethesda, MD: USAID ASSIST Project Experience Improving HIV Services; 2014 Available at: https://www.usaidassist.org/sites/assist/files/usaid_assist_experience_improving_hiv_services_ada_june2014_0.pdf. Accessed May 1, 2018.

[43] USAID. Lessons Learned from Applying Collaborative Improvement Methodologies to Strengthen the Performance and Productivity of HIV Human Resources—Technical Report. Washington, DC: USAID; 2016.

[44] Batalden PB Davidoff F What is quality improvement; and how can it transform healthcare? Qual Saf Health Care. 2007;16:2–3. 17301192 10.1136/qshc.2006.022046 PMC2464920

[45] Massoud MR Kimble LE Goldmann D Salzburg global seminar session 565—“Better health care: how do we learn about improvement?” Int J Qual Heal Care. 2018;30:1–4. 10.1093/intqhc/mzy020 PMC5909659 29447364

[46] Kågesten AE Tunçalp Ö Portela A Programme Reporting Standards (PRS) for improving the reporting of sexual, reproductive, maternal, newborn, child and adolescent health programmes. BMC Med Res Methodol. 2017;17:117. 28774287 10.1186/s12874-017-0384-7 PMC5543449

[47] Barry D Kimble L Nambiar B A framework for learning about improvement: embedded implementation and evaluation design to optimize learning. Int J Qual Heal Care. 2018;30:10–14. 10.1093/intqhc/mzy008 PMC5909667 29873794

[48] Bardfield J Palumbo M Geis M; NOA Working Group T. A National Organizational Assessment (NOA) to build sustainable quality management programs in low- and middle-income countries. Jt Comm J Qual Patient Saf. 2016;42:325–330. 27301837 10.1016/s1553-7250(16)42045-3

[49] Opondo C Allen E Todd J Association of the Paediatric Admission Quality of Care score with mortality in Kenyan hospitals: a validation study. Lancet Glob Heal. 2018;6:e203–10. 10.1016/S2214-109X(17)30484-9 PMC5785367 29389541

[50] SQUIRE. SQUIRE | SQUIRE 2.0 Guidelines. 2017 Available at: http://www.squire-statement.org/index.cfm?fuseaction=page.viewpage&pageid=471. Accessed March 27, 2018.

[51] Johnston S Kendall C Hogel M Measures of quality of care for people with HIV: a scoping review of performance indicators for primary care. PLoS One. 2015;10:e0136757. 26414994 10.1371/journal.pone.0136757 PMC4586139

[52] Adeniran A Likaka A Costello A Leadership, action, learning and accountability to deliver quality care for women, newborns and children. Bull World Heal Organ. 2018;96:222–224. 10.2471/BLT.17.197939 PMC5840625 29531422

[53] World Health Organization. Chapter 11—Quality Improvement (QI): Introduction to Quality Improvement. Available at: http://www.who.int/hiv/pub/imai/om_11_quality_improvement.pdf. Accessed February 21, 2018.

[54] World Health Organization. Quality of Care—A Process for Making Strategic Choices in Health Systems. 2006 Available at: http://www.who.int/management/quality/assurance/QualityCare_B.Def.pdf. Accessed February 21, 2018.

[55] Barker PM Reid A Schall MW A framework for scaling up health interventions: lessons from large-scale improvement initiatives in Africa. Implement Sci. 2015;11:12. 10.1186/s13012-016-0374-x PMC4731989 26821910

[56] Kruk ME Pate M Mullan Z Introducing the Lancet global health commission on high-quality health systems in the SDG Era. Lancet Glob Heal. 2017;5:e480–1. 10.1016/S2214-109X(17)30101-8 28302563

[57] Nambiar B Hargreaves DS Morroni C Improving health-care quality in resource-poor settings. Bull World Health Organ. 2017;95:76–78. 28053367 10.2471/BLT.16.170803 PMC5180347

[58] World Health Organization. WHO | WHO Framework on Integrated People-Centred Health Services. WHO 2018 Available at: http://www.who.int/servicedeliverysafety/areas/people-centred-care/en/. Accessed March 7, 2018.

